# The Mediating Role of Social Support in the Relationship Between Physical Activity and Mental Health Among Chinese College Students

**DOI:** 10.1192/bjo.2026.11159

**Published:** 2026-06-30

**Authors:** Xinxin Fan, Tianhu Lyu, Xiaolong Jin, Xi Hou, Shuhua Wang

**Affiliations:** The Sixth Affiliated Hospital of Kunming Medical University, Yuxi, China

## Abstract

**Aims:**

To investigate (1) the prevalence of anxiety and depression, (2) the association between physical activity (PA) and these mental health outcomes, and (3) the mediating role of social support in this relationship among college students, while controlling for gender and age.

**Methods:**

Nationwide cross-sectional online survey (March 2024–May 2025) of 1,069 college students recruited through student-union mailing lists in China (92.7% response rate, mean age=21 years, 68.2% female). PA was assessed using the International Physical Activity Questionnaire-Short Form (IPAQ-SF) and categorized into low, moderate and high levels; continuous MET-min/week scores were also used. Anxiety and depression were measured by the Zung Self-Rating Anxiety Scale (SAS) and Depression Scale (SDS), reported as binary outcomes (SAS≥50, SDS≥53) and standard scores. Social support was evaluated using the Social Support Rating Scale (SSRS). Analyses included binary logistic regression and mediation analysis using PROCESS Model 4 with 5,000 bootstrap samples, controlling for gender and age.

**Results:**

Overall, 34.0% of participants screened positive for anxiety (10.5% moderate, 5.5% severe) and 43.5% for depression (9.4% moderate, 1.0% severe). Logistic regression showed that compared to high PA, low PA was associated with significantly higher risks of anxiety (OR=2.21, 95% CI:1.58–3.09) and depression (OR=2.91, 95% CI:2.10–4.01). Higher social support was associated with lower risks of both anxiety (OR=0.95) and depression (OR=0.96). Mediation analysis revealed that social support partially mediated the PA-mental health relationship: for anxiety, total standardized effect of PA β=−0.127 (p<0.001), indirect effect through social support β=−0.044, 95% CI [−0.062,−0.028] (34.2% mediated); for depression, total effect β=−0.173 (p<0.001), indirect effect β=−0.053, 95% CI [−0.073,−0.035] (30.5% mediated). Gender and age were not significant covariates in the mediation pathways.

**Conclusion:**

This study identifies social support as a significant partial mediator in the PA-mental health relationship among college students.The mediating effect is proportionally larger for anxiety, although the association of social support is stronger for depression.These findings suggest that interventions should integrate PA promotion with social support enhancement to maximize mental health benefits.

